# Intermediate-Term Prognostic Value of Homocysteine in Acute Coronary Syndrome Complicated with or without Hypertension: A Multicenter Observational Cohort Study

**DOI:** 10.31083/j.rcm2407210

**Published:** 2023-07-18

**Authors:** Qiang Chen, Shiqiang Xiong, Xunshi Ding, Xiuqiong Yu, Caiyan Cui, Hong Su, Yu Long, Yan Luo, Zhen Zhang, Hanxiong Liu, Tao Xiang, Lin Cai

**Affiliations:** ^1^Department of Cardiology, the Third People's Hospital of Chengdu, Affiliated Hospital of Southwest Jiaotong University, 610014 Chengdu, Sichuan, China; ^2^Department of Cardiology, Sichuan Mianyang 404 Hospital, 621053 Mianyang, Sichuan, China; ^3^Department of Emergency, the Third People's Hospital of Chengdu, Affiliated Hospital of Southwest Jiaotong University, 610014 Chengdu, Sichuan, China

**Keywords:** acute coronary syndrome, homocysteine, hypertension, prognosis

## Abstract

**Background:**

As a classical biomarker associated with hypertension, the prognostic value of homocysteine (Hcy) in the intermediate-term outcome of acute coronary syndrome (ACS) remains controversial. This study aimed to investigate the role of homocysteine in ACS patients with different blood pressure statuses.

**Methods:**

A total of 1288 ACS patients from 11 general hospitals in Chengdu, China, from June 2015 to December 2019 were consecutively included in this observational study. The primary endpoint was defined as all-cause death. Secondary endpoints included cardiac death, nonfatal myocardial infarction (MI), unplanned revascularization and nonfatal stroke. The patients in the hypertension group (n = 788) were further stratified into hyperhomocysteinemia (H-Hcy, n = 245) and normal homocysteinaemia subgroups (N-Hcy, n = 543) around the cut-off value of 16.81 µmol/L. Similarly, the nonhypertensive patients were stratified into H-Hcy (n = 200) and N-Hcy subgroups (n = 300) around the optimal cut-off value of 14.00 µmol/L. The outcomes were compared between groups.

**Results:**

The median follow-up duration was 18 months. During this period, 78 (6.05%) deaths were recorded. Kaplan‒Meier curves illustrated that H-Hcy had a lower survival probability than N-Hcy in both hypertension and nonhypertension groups (*p* < 0.01). Multivariate Cox regression analysis revealed that H-Hcy was a predictor of intermediate-term mortality in ACS, regardless of blood pressure status.

**Conclusions:**

Elevated Hcy levels predict intermediate-term all-cause mortality in ACS regardless of blood pressure status. This association could be conducive to risk stratification of ACS.

**Clinical Trial Registration:**

The study was registered in the Chinese Clinical Trials Registry in China (ChiCTR1900025138).

## 1. Introduction 

Acute coronary syndrome (ACS) remains a serious type of coronary atherosclerotic disease (CAD) with high morbidity and mortality worldwide [1]. Despite receiving the optimum treatment recommended by modern guidelines, including early revascularization of lesions, dual antiplatelet treatment and intensive lipid-lowering therapy, some ACS patients are still at risk for recurrence of adverse cardiovascular events. Identifying high-risk ACS populations based on prognostic risk factors, including cardiometabolic factors, and providing them with optimal comprehensive treatment and nursing care is necessary to further improve their prognosis [2].

Homocysteine (Hcy), derived from methionine (Met) metabolism, along with uric acid, proinflammatory molecules (such as C-reactive protein), glucose metabolism, dyslipidemia, overweight or obesity and hypertension, has received attention as a newly emerging cardiometabolic risk factor for cardiovascular disease (CVD) [3] by promoting plaque formation and atherosclerosis, causing platelet aggregation and blood coagulation, altering lipid metabolism, and triggering inflammatory responses [4]. Previous studies have illustrated that elevated serum Hcy was associated with higher risks of cardiovascular events in ACS patients [5, 6, 7]. In contrast, a Mendelian randomization study indicated that there is no causal relationship between plasma Hcy and CVD or acute myocardial infarction (AMI) [8]. Thus, the conflicting findings from current studies render the relationship between Hcy and the outcome of ACS controversial.

In addition, an elevated Hcy level is strongly associated with the occurrence and progression of hypertension by inhibiting endogenous hydrogen sulfide generation and activating angiotensin-converting enzymes [9, 10]. Previous studies have reported that hypertension and hyperhomocysteinemia have a significant synergistic effect on the prognosis of CVD [11, 12]. Hcy could have a different influence on prognosis in ACS patients with or without hypertension. However, most studies currently adopt the definite Hcy classification criteria for all ACS patients to guide risk stratification, regardless of their blood pressure status, which might misestimate their actual risk. Therefore, our study intended to adopt different cut-off values determined by receiver operating characteristic (ROC) curve analysis in hypertensive and nonhypertensive patients with ACS to explore the prognostic significance of Hcy in the intermediate-term outcomes of patients with different blood pressure statuses.

## 2. Materials and Methods

### 2.1 Study Population and Design

A total of 1288 ACS patients from 11 general hospitals in Chengdu from June 2015 to December 2019 were consecutively included in this observational study. The diagnosis of ACS, including ST-elevation myocardial infarction (STEMI), non-ST-elevation myocardial infarction (NSTEMI), and unstable angina pectoris (UA), was guided by the corresponding guidelines [13, 14]. The exclusion criteria were as follows: (1) age younger than 18 years; (2) incomplete baseline data; (3) loss to follow-up; (4) uncompensated chronic renal dysfunction with creatinine clearance (CrCl) <15 mL/min; (5) complicated severe chronic disease with a life expectancy <1 year; and (6) death in hospital.

**Fig. 1.**
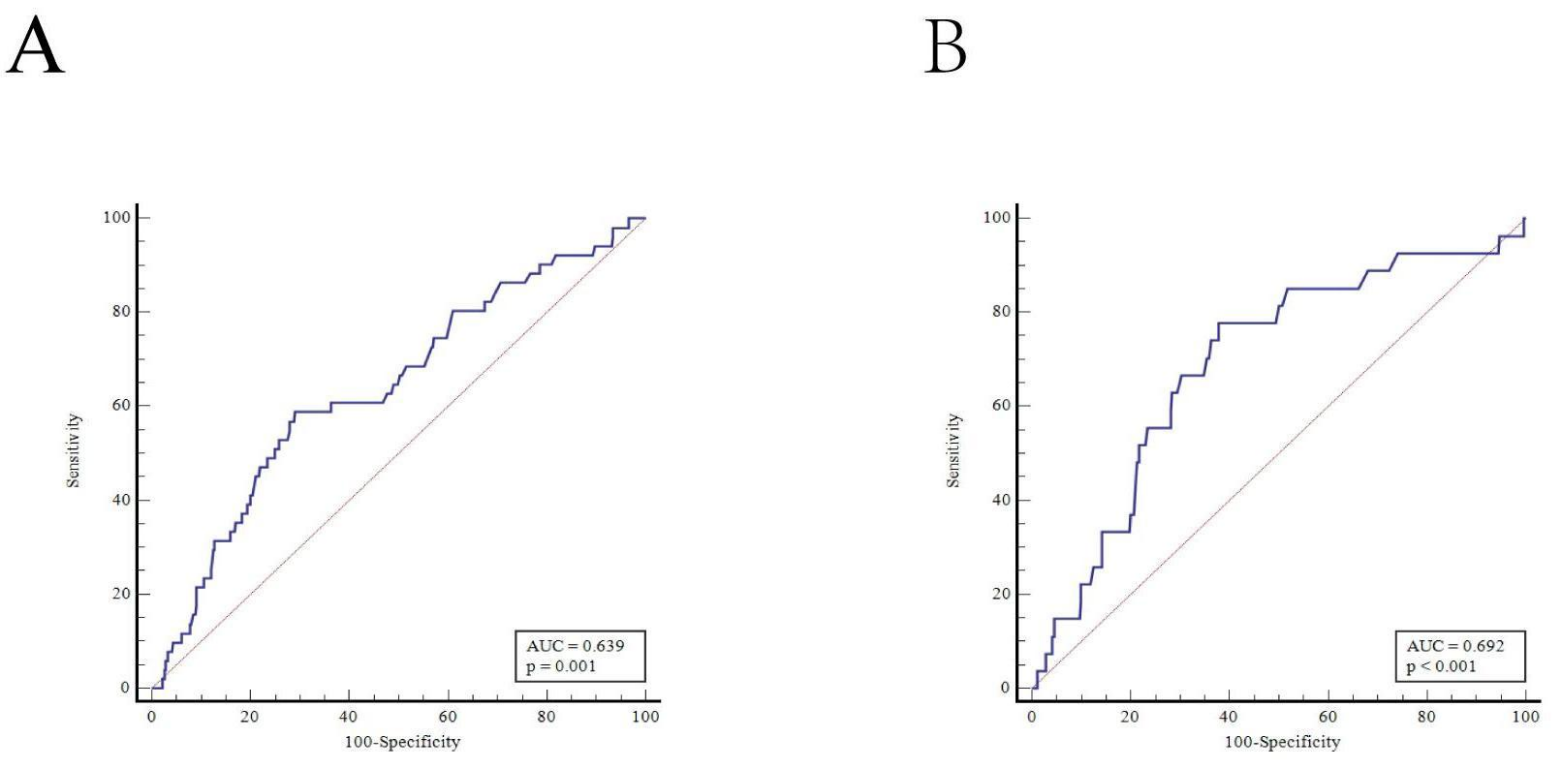
**ROC curve analysis determined the optimum cut-off value of plasma homocysteine concentration to measure intermediate-term mortality**. (A) ROC curve analysis in hypertension. (B) ROC curve analysis in nonhypertension. AUC, area under the ROC curve; ROC, receiver operating characteristic.

The demographic, clinical, biochemical, and angiographic data and discharge medications were gathered by trained professionals from the hospital medical records system. Patients were classified into two groups based on their discharge diagnosis: hypertension and nonhypertension. To further increase the prognostic importance of the study, the optimum cut-off value for plasma Hcy concentration to measure intermediate-term mortality was evaluated by ROC curve analysis. According to the optimum cut-off value of each group, hypertensive patients were further subdivided into hyperhomocysteinemia (H-Hcy) (n = 245) and normal homocysteinemia (N-Hcy) groups (n = 543) (the area under the ROC curve (AUC) was 0.639, the sensitivity was 58.8%, the specificity was 71.0%, and the optimal cut-off value was 16.81 µmol/L, *p* < 0.001, Fig. 1A). Similarly, nonhypertensive patients were subdivided into H-Hcy (n = 200) and N-Hcy groups (n = 300) (the AUC was 0.692, the sensitivity was 77.8%, the specificity was 62.2%, and the optimal cut-off value was 14.0 µmol/L, *p* < 0.001, Fig. 1B).

The study was registered in the Chinese Clinical Trials Registry in China (ChiCTR1900025138). We affirm that our protocol was conducted in compliance with the Declaration of Helsinki and approved by the local ethics committee. Due to its retrospective nature, the committee waived the requirement for formal informed consent.

### 2.2 Follow-up and Definitions

After discharge, regular follow-up was performed by a professional cardiologist at 1, 3, 6, and 12 months and then annually thereafter. Prognostic information was obtained by consulting electronic medical records or telephone inquiries. We discontinued follow-up when death was recorded. The primary endpoint was defined as all-cause death. The secondary endpoints included cardiac death, nonfatal myocardial infarction (MI), unplanned revascularization, and nonfatal stroke.

Hypertension was defined as a systolic blood pressure (SBP) ≥140 mmHg and/or a diastolic blood pressure (DBP) ≥90 mmHg during hospitalization or a history of hypertension [15]. Premature ACS referred to the occurrence of ACS in men younger than 55 years old and women younger than 65 years old [16]. Multivessel disease meant stenosis >50% in >1 of the major coronary arteries [17].

Cardiac death meant death driven by MI, heart failure (HF) and/or arrhythmia and included sudden death without a definite cause [18]. Unplanned revascularization meant the recurrent revascularization of any lesion by percutaneous coronary intervention (PCI) or coronary artery bypass grafting (CABG) [19]. Stroke was defined as ischaemic or hemorrhagic stroke during the follow-up period as confirmed by imaging and diagnosed by professional neurologists.

### 2.3 Statistical Analysis

Continuous data are expressed as the mean ± standard deviation (SD) or interquartile range (IQR). They were compared using Student’s *t*-test or the Mann‒Whitney U test. Categorical variables are expressed as percentages and were compared using the chi-square test or Fisher’s exact test. The optimum cut-off value of serum Hcy was obtained from ROC curve analysis. The time-to-event data were plotted using the Kaplan‒Meier method, and the log-rank test was applied to evaluate discrepancies between the groups. With all-cause death as the dependent variable, univariate Cox analysis was conducted. Then, multivariate Cox analysis was done to evaluate whether elevated Hcy concentrations were linked to a worse prognosis. MedCalc Statistical Software, version 19.6.1 (MedCalc Software, Ostend, Belgium), was used for all statistical analyses. All statistical tests were 2-tailed, and a *p* value < 0.05 was considered to be statistically significant.

## 3. Results

### 3.1 Baseline Characteristics

This analysis included 1288 ACS patients (595 UA, 396 STEMI, and 297 NSTEMI), including 788 hypertensive patients (61.2%) and 500 nonhypertensive patients (38.8%), with an average age of 66.58 ± 12.12 years. The median plasma Hcy level was 13.82 (IQR, 11.04–18.44) µmol/L in the hypertension group and 12.85 (IQR, 10.4–16.5) µmol/L in the nonhypertension group. The baseline characteristics stratified by different blood pressure statuses are presented in Table 1. The hypertension group was older; included more women; had a higher prevalence of diabetes mellitus, stroke history, multivessel disease and calcified lesions; and had higher levels of SBP, serum creatinine, uric acid, and plasma Hcy (*p* < 0.05). However, their proportions of current smokers, thrombosis, and AMI were lower than those in nonhypertensive patients (*p* < 0.05). Regarding discharge medications, there were significant differences in the use of angiotensin converting enzyme inhibitor/angiotensin II receptor blocker (ACEIs/ARBs) and diuretics between the patients with and without hypertension.

**Table 1.** **Baseline characteristics of the study patients stratified by blood pressure status**.

Variable		Total population	Hypertension (n = 788)	Non-hypertension (n = 500)	*p*
Age, years		66.58 ± 12.12	68.75 ± 11.16	63.17 ± 12.79	<0.001
Female, n (%)		358 (27.8)	267 (33.9)	91 (19.2)	<0.001
Smoking, n (%)		492 (38.2)	254 (32.2)	238 (47.6)	<0.001
Previous PCI, n (%)		112 (8.7)	71 (9.0)	41 (8.2)	0.615
Previous stroke, n (%)		72 (5.6)	59 (7.5)	13 (2.6)	<0.001
Diabetes mellitus, n (%)		406 (31.5)	290 (36.8)	116 (23.2)	<0.001
SBP, mmHg		132.78 ± 22.82	138.13 ± 23.62	124.42 ± 18.68	<0.001
HR, bpm		77.72 ± 15.91	76.92 ± 15.12	78.97 ± 17.04	0.024
cTnT, pg/mL		24.65 (9.88, 434.33)	22.17 (10.34, 289.00)	33.96 (8.47, 1042.5)	0.109
BNP, pg/mL		135.80 (66.95, 429.18)	131.75 (67.13, 429.18)	139.35 (62.25, 429.18)	0.857
Creatinin, µmol/L		77.05 (65.03, 92.40)	79.85 (65.80, 96.00)	73.95 (64.50, 87.93)	<0.001
Uric acid, µmol/L		378.44 ± 114.76	387.00 ± 120.79	364.94 ± 103.27	0.001
FBG, mmol/L		7.31 ± 3.69	7.42 ± 3.95	7.15 ± 3.22	0.202
Triglyceride, mmol/L		1.40 (1.02, 2.13)	1.44 (1.03, 2.18)	1.37 (1.00, 1.98)	0.132
Total cholesterol, mmol/L		4.37 (3.62, 5.18)	4.31 (3.58, 5.20)	4.40 (3.43, 5.15)	0.617
LDL-C, mmol/L		2.64 (2.09, 3.28)	2.59 (2.05, 3.28)	2.71 (2.19, 3.28)	0.064
HDL-C, mmol/L		1.12 (0.95, 1.34)	1.14 (0.96, 1.35)	1.11 (0.94, 1.33)	0.532
Lp (a), mg/L		153.20 (66.23, 276.15)	138.8 (65.7, 267.88)	173.5 (69.63, 308.43)	0.148
Hcy, µmol/L		13.40 (10.70, 17.59)	13.83 (11.04, 18.44)	12.85 (10.40, 16.50)	0.001
H-Hcy, n (%)		445 (34.5)	245 (31.1)	200 (40.0)	0.001
Multivessel disease, n (%)		732 (56.8)	469 (59.5)	263 (52.6)	0.015
Calcified lesions, n (%)		138 (10.7)	106 (13.5)	32 (6.4)	<0.001
Thrombosis, n (%)		49 (3.8)	22 (2.8)	27 (5.4)	0.017
LVEF		55.18 ± 9.50	55.25 ± 9.45	55.05 ± 9.59	0.715
LVEF <40 (%)		95 (7.5)	60 (7.6)	36 (7.2)	0.783
Premature ACS (%)		291 (22.6)	144 (18.3)	147 (29.4)	<0.001
AMI, n (%)		693 (53.8)	389 (49.4)	304 (60.8)	<0.001
Diagnosis, n (%)					<0.001
	UA	595 (46.2)	399 (50.6)	196 (39.2)	
	NSTEMI	297 (23.1)	185 (23.5)	112 (22.4)	
	STEMI	396 (30.7)	204 (25.9)	192 (38.4)	
PCI, n (%)		1102 (85.6)	674 (85.5)	428 (85.6)	0.973
Discharge medications					
	Aspirin, n (%)	1224 (95.0)	756 (95.9)	468 (93.6)	0.060
	P2Y12 receptor inhibitor, n (%)	1265 (98.2)	778 (98.7)	487 (97.4)	0.079
	Statins, n (%)	1231 (95.6)	750 (95.2)	481 (96.2)	0.385
	β-blockers, n (%)	893 (69.3)	547 (69.4)	346 (69.2)	0.935
	ACEI/ARB, n (%)	584 (45.3)	458 (58.1)	126 (25.2)	<0.001
	Diuretics, n (%)	225 (17.5)	162 (20.6)	63 (12.6)	<0.001

Note: 1 mmHg = 0.133 kPa. Abbreviations: cTnT, troponin T; BNP, B-type natriuretic peptide; UA, unstable angina; LDL-C, low-density lipoprotein; HDL-C, high-density lipoprotein; FBG, fasting blood glucose; Hcy, homocysteine; H-Hcy, hyperhomocysteinemia; LVEF, left ventricular ejection fraction; ACEI/ARB, angiotensin-converting enzyme inhibitor/angiotensin receptor blocker; PCI, percutaneous coronary intervention; SBP, systolic blood pressure; AMI, acute myocardial infarction; NSTEMI, non-ST-elevation myocardial infarction; STEMI, ST-elevation myocardial infarction; HR, heart rate; Lp (a), lipoprotein (a).

Table 2 summarizes the baseline demographic, clinical, biochemical, and angiographic data of hypertension and nonhypertension groups when stratified by the cut-off value for plasma Hcy. H-Hcy subjects were older and had higher levels of serum B-type natriuretic peptide (BNP), creatinine, and uric acid and lower left ventricular ejection fraction (LVEF) in both hypertension and nonhypertension groups (*p* < 0.05 for both). In addition, the proportions of patients with a history of stroke and the use of diuretics were higher in the population with H-Hcy in both groups (*p* < 0.05 for both). In the hypertension group, the H-Hcy subgroup had fewer women and lower SBP, heart rate (HR), low-density lipoprotein cholesterol (LDL-C) and high-density lipoprotein cholesterol (HDL-C) levels (*p* < 0.05) than the N-Hcy group. Moreover, the proportion of heart failure (LVEF <40%) in the subgroup with H-Hcy was higher (*p* < 0.05). In the nonhypertension group, H-Hcy patients had a larger proportion of calcified coronary lesions, and these patients had a lower level of total cholesterol (*p* < 0.05). Additionally, smoking habit, previous revascularization therapy, diabetes mellitus, multivessel disease, and the levels of triglycerides and Lp(a) did not differ between hypertension and nonhypertension subgroups (*p* > 0.05).

**Table 2.** **Baseline characteristics of patients in different Hcy subgroups**.

Variable		Hypertension (n = 788)			Non-hypertension (n = 500)		
		H-Hcy (n = 245)	N-Hcy (n = 543)	*p*	H-Hcy (n = 200)	N-Hcy (n = 300)	*p*
Age, years		70.82 ± 11.08	67.82 ± 11.08	<0.01	65.52 ± 14.53	61.6 ± 11.23	<0.01
Female, n (%)		55 (22.4)	212 (39.0)	<0.01	31 (15.5)	60 (20.0)	0.20
Smoking, n (%)		78 (31.8)	176 (32.4)	0.873	92 (46)	146 (48.7)	0.56
previous PCI, n (%)		24 (9.8)	47 (8.7)	0.605	19 (9.5)	22 (7.3)	0.387
Previous stroke, n (%)		29 (11.8)	30 (5.5)	<0.01	10 (5)	3 (1)	<0.01
Diabetes mellitus, n (%)		92 (37.6)	198 (36.5)	0.77	35 (17.5)	81 (27)	0.014
SBP, mmHg		134.35 ± 23.42	139.72 ± 23.30	0.003	125.76 ± 19.22	123.61 ± 18.22	0.208
HR, bpm		74.87 ± 15.42	77.84 ± 14.90	0.011	80.34 ± 18.45	78.06 ± 15.96	0.143
cTnT, pg/mL		28.13 (13.62, 285.90)	19.71 (8.77, 290.00)	0.013	37.94 (8.43, 1231.00)	33.96 (8.47, 730.70)	0.876
BNP, pg/mL		175.2 (78, 577.1)	120.4 (59.2, 378.5)	<0.01	170.85 (68, 435.49)	130.9 (59.25, 365.45)	0.028
Creatinin, µmol/L		96 (80.15, 126.5)	74.7 (62.9, 87.6)	<0.01	79.95 (68.27, 95.5)	70.9 (62.37, 81.47)	<0.01
Uric acid, µmol/L		441.93 ± 130.38	362.22 ± 107.42	<0.01	397.24 ± 122.47	343.41 ± 81.57	<0.01
FBG, mmol/L		7.21 ± 3.45	7.36 ± 3.88	0.602	7.34 ± 3.74	7.31 ± 3.50	0.919
Triglyceride, mmol/L		1.41 (1.01, 2.13)	1.45 (1.04, 2.2)	0.358	1.33 (0.97, 1.99)	1.38 (1, 1.97)	0.873
Total cholesterol, mmol/L		4.3 (3.51, 5.11)	4.32 (3.6, 5.23)	0.323	4.29 (3.51, 4.99)	4.47 (3.84, 5.2)	0.027
LDL-C, mmol/L		2.44 (1.94, 3.08)	2.63 (2.06, 3.34)	0.014	2.65 (2.1, 3.25)	2.76 (2.24, 3.34)	0.14
HDL-C, mmol/L		1.09 (0.9, 1.31)	1.14 (0.97, 1.37)	<0.01	1.11 (0.94, 1.35)	1.1 (0.94, 1.32)	0.543
Lp (a), mg/L		133 (60.1, 265.52)	144 (67.9, 273.3)	0.405	189.6 (76.9, 266.55)	163.35 (65.77, 327.75)	0.644
Multivessel disease, n (%)		150 (61.2)	319 (58.7)	0.512	110 (55)	153 (51)	0.38
Calcified lesions, n (%)		36 (14.7)	70 (12.9)	0.492	19 (9.5)	13 (4.3)	0.021
Thrombosis, n (%)		7 (2.9)	15 (2.8)	0.940	9 (4.5)	18 (6.0)	0.467
LVEF		53.44 ± 10.17	56.06 ± 8.99	<0.01	53.45 ± 9.77	56.12 ± 9.33	<0.01
LVEF <40 (%)		28 (11.4)	32 (5.9)	<0.01	17 (8.5)	19 (6.3)	0.359
Premature ACS (%)		29 (11.8)	115 (21.2)	<0.01	49 (24.5)	98 (32.7)	0.05
AMI, n (%)		133 (54.3)	256 (47.1)	0.064	125 (62.5)	179 (59.7)	0.525
PCI, n (%)		205 (83.7)	469 (86.4)	0.319	166 (83.0)	262 (87.3)	0.176
Diagnosis, n (%)				0.104			0.516
UA		112 (45.7)	287 (52.9)		75 (37.5)	121 (40.3)	
NSTEMI		68 (27.8)	117 (21.5)		50 (25.0)	62 (20.7)	
STEMI		65 (26.5)	139 (25.6)		75 (37.5)	117 (39)	
Discharge medications							
	Aspirin, n (%)	236 (96.3)	520 (95.8)	0.711	183 (91.5)	285 (95.0)	0.117
	$P_{2}$$Y_{12}$ receptor inhibitor, n (%)	242 (98.8)	53.6 (98.7)	0.940	193 (96.5)	294 (98.0)	0.302
	Statins, n (%)	237 (96.7)	513 (94.5)	0.171	191 (95.5)	290 (96.7)	0.504
	β-blockers, n (%)	159 (64.9)	388 (71.5)	0.064	134 (67.0)	212 (70.7)	0.384
	ACEI/ARB, n (%)	133 (54.3)	325 (59.9)	0.143	62 (31.0)	64 (21.3)	0.015
	Diuretics, n (%)	65 (26.5)	97 (17.9)	0.005	34 (17.0)	29 (9.7)	0.015

Note: 1 mmHg = 0.133 kPa. Abbreviations: cTnT, troponin T; BNP, B-type natriuretic peptide; UA, unstable angina; HR, heart rate; LDL-C, low-density lipoprotein cholesterol; HDL-C, high-density lipoprotein cholesterol; N-Hcy, normal homocysteinemia; FBG, fasting blood glucose; Hcy, homocysteine; H-Hcy, hyperhomocysteinemia; LVEF, left ventricular ejection fraction; ACEI/ARB, angiotensin converting enzyme inhibitor/angiotensin II receptor blocker; SBP, systolic blood pressure; AMI, acute myocardial infarction; PCI, percutaneous coronary intervention; STEMI, ST-elevation myocardial infarction; NSTEMI, non-ST-elevation myocardial infarction; Lp (a), lipoprotein (a).

### 3.2 Intermediate-Term Clinical Outcomes 

**Fig. 2.**
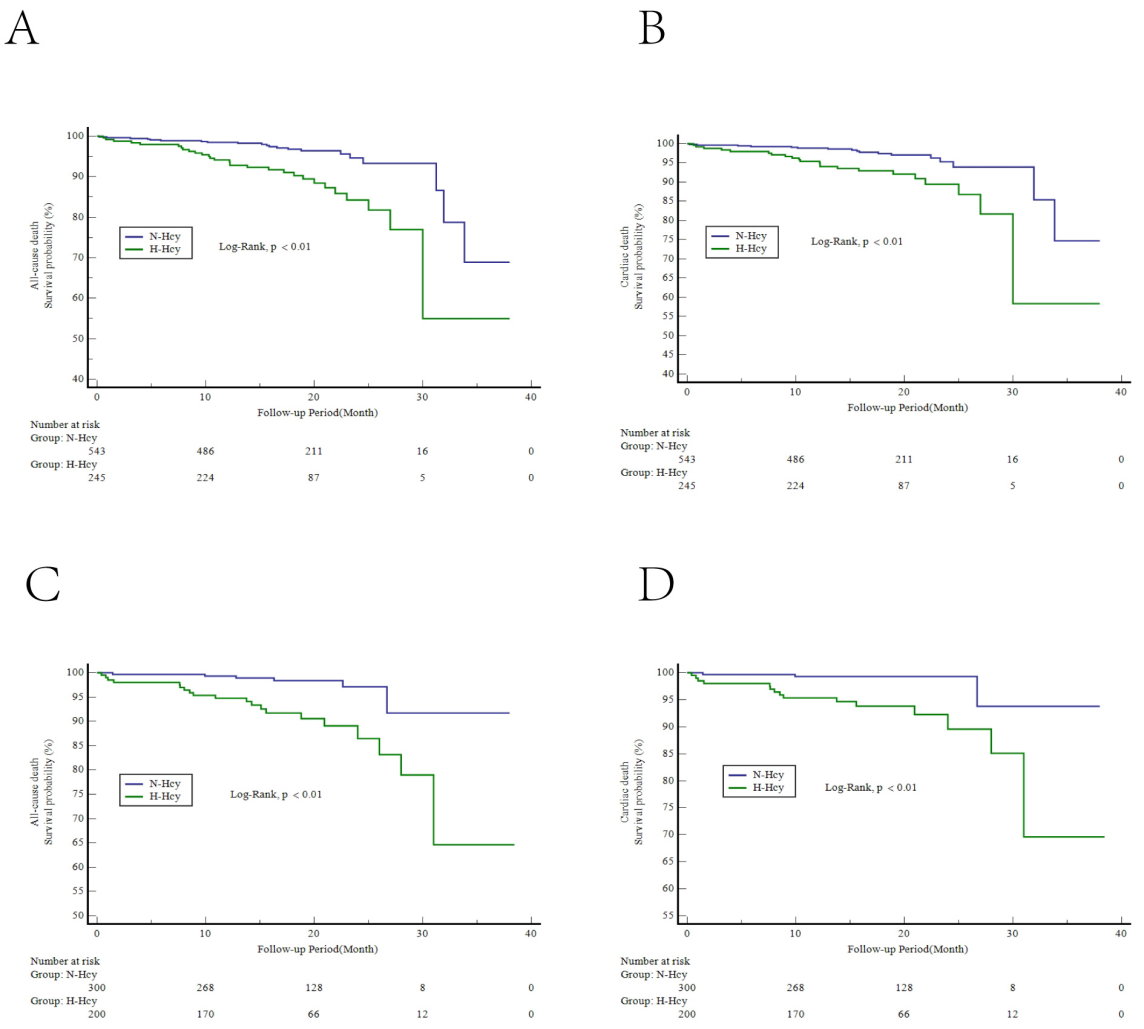
**Kaplan-Meier curves of intermediate-term clinical outcomes**. Kaplan-Meier curves for the survival probability of all-cause death (A) and cardiac death (B) in the hypertension group and all-cause death (C) and cardiac death (D) in the nonhypertension group. H-Hcy, hyperhomocysteinemia; N-Hcy, normal homocysteinemia.

The median follow-up duration was 18 (range: 13.83–22.37) months, and 78 (6.05%), 59 (4.58%), 34 (2.64%), 104 (8.07%), and 10 (0.77%) cases of all-cause death, cardiac death, nonfatal MI, revascularization, and nonfatal stroke were recorded, respectively. The number of all-cause mortality and cardiac death events was higher in the H-Hcy subgroup than in the N-Hcy subgroup of both hypertensive and nonhypertensive patients (*p* < 0.01) (Table 3). The survival analysis illustrated that the H-Hcy subgroup had a lower survival probability from all-cause death and cardiac death than the N-Hcy subgroup in both the hypertension (Fig. 2A,B) and nonhypertension groups (Fig. 2C,D) (*p* < 0.01).

**Table 3.** **Intermediate-term clinical outcomes**.

	Hypertension (n = 788)			Non-hypertension (n = 500)		
	H-Hcy (n = 245)	N-Hcy (n = 543)	*p*	H-Hcy (n = 200)	N-Hcy (n = 300)	*p*
All-cause death, n (%)	30 (12.2)	21 (3.9)	<0.01	21 (10.5)	6 (2.0)	<0.01
Cardiac death, n (%)	23 (9.4)	17 (3.1)	<0.01	16 (8.0)	3 (1.0)	<0.01
Non-fatal MI, n (%)	8 (3.3)	12 (2.2)	0.383	7 (3.5)	7 (2.3)	0.439
Unplanned revascularization, n (%)	22 (9.0)	43 (7.9)	0.616	13 (6.5)	26 (8.7)	0.376
Non-fatal stroke, n (%)	3 (1.2)	4 (0.7)	0.791	1 (0.5)	2 (0.7)	>0.99

N-Hcy, normal homocysteinemia; H-Hcy, hyperhomocysteinemia; MI, myocardial infarction.

### 3.3 Predictors of Intermediate-Term All-Cause Death 

**Fig. 3.**
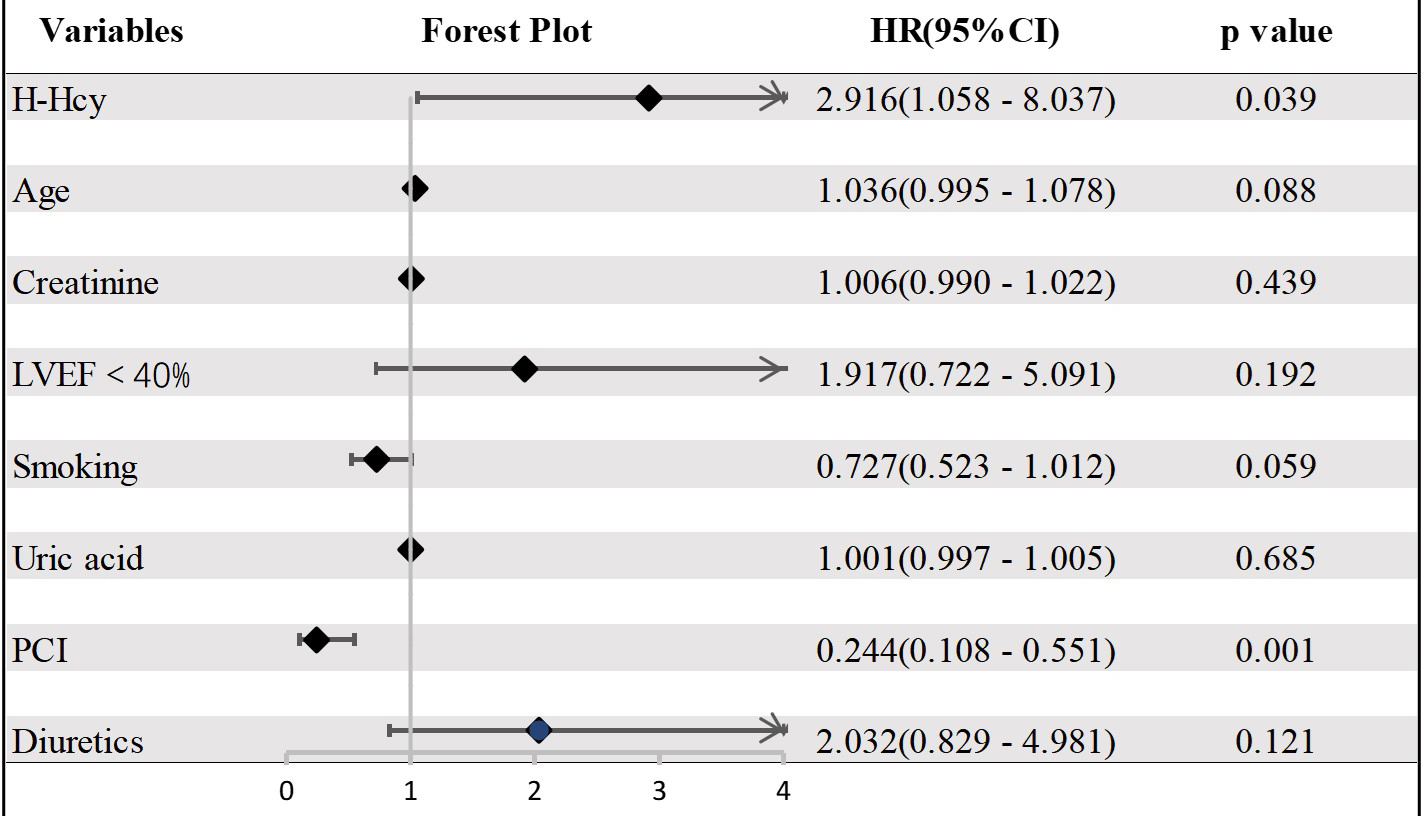
**Forest plot of all-cause death in the nonhypertension group**. CI, confidence interval; LVEF, left ventricular ejection fraction; PCI, percutaneous coronary intervention; H-Hcy, hyperhomocysteinemia; HR, hazard radio.

**Fig. 4.**
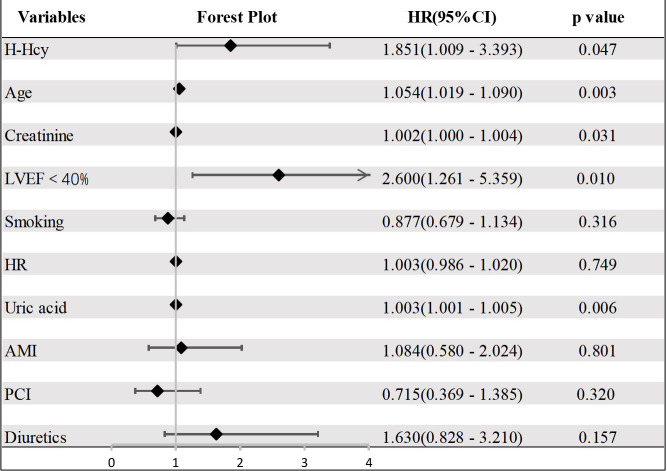
**Forest plot of all-cause death in the hypertension group**. CI, confidence interval; LVEF, left ventricular ejection fraction; PCI, percutaneous coronary intervention; H-Hcy, hyperhomocysteinemia; AMI, acute myocardial infarction; HR, heart rate.

The univariate analysis results are presented in **Supplementary Table 1**. In the nonhypertension group, H-Hcy, age, creatinine, LVEF <40%, smoking, uric acid, diuretics, and PCI were relevant to the risk of all-cause death. After adjusting for confounding factors, multivariate Cox analysis indicated that H-Hcy was an independent predictor of all-cause death (HR 2.916, 95% CI: 1.058 to 8.037, *p* = 0.039) (Fig. 3, **Supplementary Table 2**). Conversely, in the hypertension group, multivariate Cox regression analysis implied that the independent predictors of all-cause death were age (HR = 1.054, 95% CI: 1.019 to 1.090, *p* = 0.003), H-Hcy (HR = 1.851, 95% CI: 1.009 to 3.393, *p* = 0.047), creatinine (HR = 1.002, 95% CI: 1.000 to 1.004, *p* = 0.031), LVEF <40% (HR = 2.600, 95% CI: 1.261 to 5.359, *p* = 0.010), and uric acid (HR = 1.003, 95% CI: 1.001 to 1.005, *p* = 0.006) (Fig. 4, **Supplementary Table 2**).

## 4. Discussion 

The results of this study revealed that (1) H-Hcy patients had higher all-cause mortality and cardiac death events than those with normal Hcy in ACS, regardless of the status of blood pressure; (2) elevated serum Hcy concentration was an independent predictor of intermediate-term all-cause mortality in ACS patients with or without hypertension; and (3) ACS patients with or without hypertension could have different thresholds of serum Hcy for predicting intermediate-term mortality, which might be conducive to optimizing the risk stratification of ACS in clinical practice.

Serum Hcy, as a classic biomarker, has been reported to be an independent risk factor for cardio-cerebrovascular diseases [3, 7, 20, 21] and is associated with plaque formation and atherosclerosis progression [4, 22] by damaging vascular endothelial cells, altering lipid metabolism, and triggering inflammatory responses. In addition, it can participate in acute coronary events by disrupting the balance between blood coagulation and fibrinolysis, leading to platelet aggregation and blood coagulation [23]. Thus, Hcy has been regarded as a prognostic factor for CAD. Li S *et al*. [24] found that H-Hcy (HR = 1.075, 95% CI: 1.032–1.120, *p* < 0.01) is an independent predictor of adverse cardiovascular and cerebrovascular events in patients with CAD who underwent drug-eluting stent implantation. A meta-analysis revealed that elevated serum Hcy in patients who underwent PCI increased the risks of all-cause mortality by an average of 3.19-fold (HR = 3.19, 95% CI: 1.90–5.34, *p* < 0.01), major adverse cardiovascular events by 1.51-fold (HR = 1.51, 95% CI: 1.23–1.85, *p* < 0.01), and cardiac death by 2.76-fold (HR = 2.76, 95% CI: 1.44–5.32, *p* < 0.01) [7].

Genetic background, eating habits, and living habits all affect the serum level of Hcy [25]. The mean Hcy levels vary between different regions or races. The threshold for H-Hcy has been inconsistent among various studies [24, 26]. Therefore, using definite cut-off values of Hcy concentrations defined by guidelines or previous classical studies to guide risk stratification might misestimate the actual risk in a given patient. In addition, because Hcy and hypertension have a synergistic effect on the prognosis of cardiovascular disease [11], Hcy could have different effects on the prognosis of ACS patients with different blood pressure statuses. Based on this fact, we divided ACS patients into hypertension and nonhypertension groups and used ROC curve analysis to determine the optimum critical value of Hcy for predicting intermediate-term mortality in ACS patients with hypertension and the critical value in those without hypertension. The two groups were then subdivided into two subgroups based on their respective optimum cut-off values: an H-Hcy subgroup and a normal Hcy subgroup. We think the research method we adopted in this study may be more reasonable than those used in other studies. The results ultimately showed that the cut-off value of Hcy for predicting intermediate-term mortality was 16.81 µmol/L in patients with hypertension and 14.0 µmol/L in patients without hypertension, which could be conducive to individualized risk stratification of ACS patients.

Kaplan‒Meier curves demonstrated that H-Hcy was associated with intermediate-term mortality, including all-cause mortality and cardiac death, during the 18-month median follow-up in the two groups, consistent with previous studies [5, 7]. After adjusting for other risk factors, multivariate Cox regression revealed that H-Hcy was strongly associated with intermediate-term mortality in both hypertensive and nonhypertensive patients. We speculate that this outcome could have the following explanations. The patients in the H-Hcy group were older and had higher levels of serum BNP, creatinine, and uric acid and a lower ejection fraction. Some of the above factors are part of the GRACE score, which is an established tool that well predicts the prognosis of ACS patients [27, 28]. Calim A* et al*. [6] recently reported a significant positive correlation between Hcy and GRACE risk score in ACS patients. Homocysteine, together with uric acid, proinflammatory molecules (represented by C-reactive protein), glucose metabolism, dyslipidemia, overweight or obesity and hypertension, are emerging cardiometabolic risk factors that could aggravate poor prognosis by resulting in systemic inflammation, oxidative stress, and ultimately the progression of atherosclerosis and CVDs [3, 29]. Although controversies exist [8], Hcy has received attention as an independent prognostic factor for CVDs.

Consistent with classical theory [30], we also found that the proportion of complicated strokes was higher in the H-Hcy group than in the N-Hcy group. A study conducted in six centers in China revealed that the risk of stroke in a high-Hcy population increased by 87% [31]. However, the study that we conducted failed to establish a link between Hcy and nonfatal stroke during the follow-up. This outcome could be due to the small sample size, short follow-up time, and few endpoints observed in this study. In addition, previous studies have shown that the use of folate can reduce the risk of stroke but not the risk of heart attack [32, 33, 34]. Patients with hyperhomocysteinemia can receive folic acid treatment early, so an increased risk of stroke in patients with hyperhomocysteinemia was not observed in this study.

Our investigation has several limitations. First, this study only explored the relationship between Hcy levels and the intermediate-term prognosis of ACS. Whether homocysteine-lowering therapy could improve the prognosis of ACS was not evaluated because critical data were not available in some centers. Second, there was inevitable bias due to the retrospective nature of this study with its relatively small sample size and relatively short follow-up duration. Third, there are differences in the ability of different hospitals to comprehensively manage and treat ACS patients, which might have influenced the observed results. Additionally, there could be discrepancies in the Hcy detection ability in different hospitals.

## 5. Conclusions

This paper suggests that elevated serum Hcy level is independently associated with all-cause mortality in ACS patients regardless of hypertension. For these patients, Hcy levels should be monitored during in-hospital stays and follow-up to help with risk stratification and management decisions, and positive and individualized interventions should be performed if necessary.

## Data Availability

The datasets used or analyzed during the current study are available from the corresponding author on reasonable request.

## Supplementary Material

Supplementary material associated with this article can be found, in the online version, at https://doi.org/10.31083/j.rcm2407210.
