# Supplementary material for: Intermediate-Term Prognostic Value of Homocysteine in Acute Coronary Syndrome Complicated with or without Hypertension: A Multicenter Observational Cohort Study
Source: Rev Cardiovasc Med. 2023 Jul 18;24(7):210. doi: 10.31083/j.rcm2407210 (PMC11266453; doi:10.31083/j.rcm2407210)
Supplement: Supplementary file 1 [file 2153-8174-24-7-210-s1.doc]

Supplementary Table 1. Univariate Cox Regression Analysis Results for All cause death during Follow-Up

| Variables | Hypertension | | |  | Non- Hypertension | | |
| --- | --- | --- | --- | --- | --- | --- | --- |
|  | HR | 95%CI | P |  | HR | 95%CI | P |
| H-Hcy | 3.1941 | 1.8268 to 5.5850 | <0.0001 |  | 4.9631 | 1.9882 to 12.3893 | 0.0006 |
| Age | 1.0829 | 1.0480 to 1.1190 | <0.0001 |  | 1.0980 | 1.0584 to 1.1390 | <0.0001 |
| female | 1.4964 | 0.8593 to 2.6056 | 0.1544 |  | 1.5126 | 0.6386 to 3.5828 | 0.3470 |
| Creatinine | 1.0019 | 1.0007 to 1.0031 | 0.0018 |  | 1.0139 | 1.0075 to 1.0203 | <0.0001 |
| LVEF＜40% | 5.4584 | 3.0336 to 9.8214 | <0.0001 |  | 3.6317 | 1.4615 to 9.0247 | 0.0055 |
| Smoking | 0.7714 | 0.6066 to 0.9808 | 0.0342 |  | 0.6733 | 0.4974 to 0.9114 | 0.0105 |
| Previous PCI | 0.9985 | 0.3598 to 2.7714 | 0.9977 |  | 2.1554 | 0.7443 to 6.2415 | 0.1569 |
| Diabetes mellitus | 1.3886 | 0.7949 to 2.4259 | 0.2487 |  | 1.3544 | 0.5668 to 3.2364 | 0.4949 |
| SBP | 1.0066 | 0.9944 to 1.0189 | 0.2915 |  | 1.0130 | 0.9953 to 1.0309 | 0.1506 |
| HR | 1.0177 | 1.0007 to 1.0350 | 0.0407 |  | 1.0152 | 0.9972 to 1.0335 | 0.0983 |
| cTnT | 1.0000 | 1.0000 to 1.0001 | 0.2457 |  | 1.0000 | 0.9998 to 1.0002 | 0.8958 |
| FBG | 1.0283 | 0.9661 to 1.0946 | 0.3800 |  | 1.0404 | 0.9510 to 1.1383 | 0.3875 |
| UA | 1.0038 | 1.0019 to 1.0057 | 0.0001 |  | 1.0042 | 1.0011 to 1.0072 | 0.0068 |
| LDL-C | 0.9611 | 0.7229 to 1.2777 | 0.7847 |  | 0.7377 | 0.4729 to 1.1508 | 0.1800 |
| HDL-C | 0.5533 | 0.2138 to 1.4320 | 0.2225 |  | 1.0870 | 0.6161 to 1.9176 | 0.7734 |
| CHO | 0.9737 | 0.7914 to 1.1979 | 0.8008 |  | 0.9429 | 0.6813 to 1.3050 | 0.7230 |
| AMI | 1.7930 | 1.0134 to 3.1723 | 0.0449 |  | 0.9311 | 0.4307 to 2.0127 | 0.8559 |
| PCI | 0.3848 | 0.2100 to 0.7051 | 0.0020 |  | 0.1804 | 0.0844 to 0.3857 | <0.0001 |
| MVD | 0.7105 | 0.4094 to 1.2330 | 0.2242 |  | 0.5003 | 0.2289 to 1.0936 | 0.0826 |
| Calcium lesion | 1.3488 | 0.6560 to 2.7732 | 0.4159 |  | 1.3101 | 0.3884 to 4.4186 | 0.6632 |
| β-blockers | 0.9631 | 0.5361 to 1.7302 | 0.9000 |  | 0.5447 | 0.2545 to 1.1658 | 0.1176 |
| Diuretics | 3.5931 | 2.0681 to 6.2426 | <0.001 |  | 4.7757 | 2.2089 to 10.3250 | 0.0001 |
| ACEI/ARB | 0.8197 | 0.4717 to 1.4245 | 0.4808 |  | 1.9186 | 0.8771 to 4.1969 | 0.1027 |

Abbreviations: AMI, acute myocardial infarction; H-Hcy, hyperhomocysteinemia; LVEF, left ventricular ejection fraction; PCI, percutaneous coronary intervention; FBG, fasting blood glucose;ACEI/ARB, Angiotensin-converting enzyme inhibitor/Angiotensin receptor blocker

Supplementary Table 2. Multivariate Cox Regression Analysis Results for All cause death during Follow-Up

| Variables | Hypertension | | | Non- Hypertension | | |
| --- | --- | --- | --- | --- | --- | --- |
|  | Adjusted HR | 95%CI | P value | HR | 95%CI | P values |
| H-Hcy | 1.851 | 1.009 to 3.393 | 0.047 | 2.916 | 1.058 to 8.037 | 0.039 |
| Age | 1.054 | 1.019 to 1.090 | 0.003 | 1.036 | 0.995 to 1.078 | 0.088 |
| Creatinine | 1.002 | 1.000 to 1.004 | 0.031 | 1.006 | 0.990 to 1.022 | 0.439 |
| LVEF＜40% | 2.600 | 1.261 to 5.359 | 0.010 | 1.917 | 0.722 to 5.091 | 0.192 |
| Smoking | 0.877 | 0.679 to 1.134 | 0.316 | 0.727 | 0.523 to 1.012 | 0.059 |
| HR | 1.003 | 0.986 to 1.020 | 0.749 | - | - | - |
| Uric acid, | 1.003 | 1.001 to 1.005 | 0.006 | 1.001 | 0.997 to 1.005 | 0.685 |
| AMI | 1.084 | 0.580 to 2.024 | 0.801 | - | - | - |
| PCI | 0.715 | 0.369 to 1.385 | 0.320 | 0.244 | 0.108 to 0.551 | 0.001 |
| Diuretics | 1.630 | 0.828 to 3.210 | 0.157 | 2.032 | 0.829 to 4.981 | 0.121 |

Abbreviations: AMI, acute myocardial infarction; H-Hcy, hyperhomocysteinemia; LVEF, left ventricular ejection fraction; PCI, percutaneous coronary intervention;
